# miRNA–mRNA integrated analysis reveals candidate genes associated with salt stress response in Halophytic *Sonneratia apetala*

**DOI:** 10.1080/15476286.2025.2496097

**Published:** 2025-04-28

**Authors:** Beibei Chen, Lishan Zhen, Zhuanying Yang, Tingting Liu, Shaoxia Yang, Wei Mu, Xiao Xiao, Jinhui Chen

**Affiliations:** a Guangdong Engineering Technology Research Center of Tropical Crops High Efficient Production, College of Coastal Agricultural Sciences, Guangdong Ocean University, Zhanjiang, PR, China; b School of Chemistry and Environment, Guangdong Ocean University, Zhanjiang, PR, China; c School of Breeding and Multiplication (Sanya Institute of Breeding and Multiplication), Hainan University, Sanya, PR, China

**Keywords:** Sonneratia apetala, third-generation sequencing, transcriptome analysis, microRNA, target gene, salt stress

## Abstract

*Sonneratia apetala* is a pioneering species of mangrove plants, which has evolved various mechanisms to tolerate salt-stress due to their long-term exposure to a salinized environment as compared to the of terrestrial freshwater plants. However, limited attempt has been made to uncover the underlying molecular mechanism of their saline adaptation. Here, we integrated mRNA and microRNA (miRNA) sequencing to identify the genes and pathways that may be involved in salt stress-response in the roots of *S. apetala*. A comprehensive full‑length transcriptome containing 295,501 high‑quality unigenes was obtained by PacBio sequencing technology. Of these, 6,686 genes exhibited significantly differential accumulation after salt stress treatment (*p* < 0.001, *Q* < 0.01). They were mainly implicated in plant signal transduction and diverse metabolic pathways, such as those involving phenylpropanoid biosynthesis, plant-pathogen interaction and protein processing. Also, our results identified the regulatory interaction between miRNA-target counterparts during salt stress. Taken together, we present the first global overview of the transcriptome of *S. apetala* roots, and identify potentially important genes and pathways associated with salt tolerance for further investigation. This study is expected to deliver novel insights in understanding the regulatory mechanism in *S. apetala* response to salt stress.

## Introduction

Salinity, which is a severe environmental problem that affects global agriculture and ecology, inhibits plant growth and development [1]. Elucidating the physiological and molecular basis of plant response would help us to identify candidates for genetic engineering of salt-tolerant varieties, and to alleviate the adverse effects of soil salinization. So, there has been a long-standing interest in this area, where numerous attempts have been devoted to uncovering the genetic mechanism that govern plants’ adaptations to saline environments. Generally, the plant response to salt stress encompass complex processes involving multiple genes, multi-signal pathways and polygene products. They are tailored at enhancing the ability of plants to maintain ion balance, osmotic balance, reactive oxygen balance and regulate metabolites that play a very important role in the salt tolerance [2]. Further, plant salt-resistant genes can be divided into two categories on the basis of their functions: one is the category of transcription factors (TFs) that regulate gene expression and signal transduction in response to salt stress, such as AP2-EREBP, WRKY, MYB, bHLH, ZFP and other TFs family members [2]. The second category is of the salt-induced genes that code for functional proteins that enhance plant salt tolerance, such as the active oxygen scavenging gene POD, SUS osmoregulatory gene, antioxidant protective agent Chitinase [3]. Notably, a third category of non-coding RNA (ncRNA)s, such as microRNAs (miRNAs) have proved to be vital regulators of salt stress response [4].

Mangroves are a community of woody plants that grow in tropical and sub-tropical estuarine intertidal zones. As a special ecosystem extending from land towards the ocean, mangroves play an important ecological role in wind and waves prevention, water purification and coastal protection [5]. However, due to the dual influence of human activities and climate change, the area of mangrove wetland has drastically reduced [6]. Also, salinity is one of the most important factors affecting the health, distribution, growth and productivity of mangroves [7,8]. With continued exposure to periodic seawater erosion and high salinization of soil, mangroves plants have evolved a special salt-tolerance mechanism that differs from that of terrestrial freshwater plants. Although mangroves can survive under a wide range of low to moderate salinity conditions [8], higher salt levels could damage their morphological structures and limit their growth [7]. The exploration of salt-tolerance mechanism of mangroves could help in identifying regulators, which could facilitate the development of varieties for high-salinity adaptation and restoration of mangrove wetland ecosystem. However, studies on mangrove salt tolerance have mostly focused on ecological and physiological aspects [9], and limited attention was paid towards the molecular mechanism of their salt-tolerance.

Various differentially expressing genes have been functionally characterized against salt stress. For example, the antisense expression of an acyl-lipid omega-3 desaturase (FDA), reduced tobacco’s resistance to salinity [10]. LrKUP8, a potassium transporter in *Lycium ruthenicum*, was up-regulated under excessive saline stress and conferred resistance when transiently expressed in tobacco [11]. Similarly, the alterations in the levels of microRNAs affect adaptive responses to salt stress [12]. In tomato, overexpression of Sly-miR398b could increase salt sensitivity, probably by regulating the antioxidant system and photosynthesis [13]. Also, over-expression miR414c, which decreased the expression of an iron superoxide dismutase gene, *GhFSD1*, which ultimately increased cotton’s sensitivity to salinity stress [14]. Such studies highlighted that salt stress response is governed by change in the expression of related genes. Additionally, it demonstrated that genome-wide analysis across control and salinity treatments is an effective way to probe the involvement of key genetic modulators and pathways that mount a tolerance response.

RNA-Seq, also known as transcriptome sequencing, has become a powerful tool for the genome-wide analysis of differentially expressed genes (DEGs) during the process of stress-response [15,16]. RNA-seq, with the help of second-generation deep sequencing technologies has enabled the determination of sequences of messenger RNA (mRNA) and non-coding RNA (ncRNA), the structure of transcribed genes, and the quantification of individual transcripts under different biological conditions. However, short reads do not provide full coverage of the entire transcripts and require very large computational assemblies, which limit the accuracy of transcript prediction [17]. Third-generation PacBio sequencing technology (Iso-Seq) has been reported to generate full-length transcripts, which helps in generating more accurate genetic information [17]. PacBio sequencing is highly suitable for de novo transcriptomic analysis of plants [18]. Currently, an effective approach for critical gene identification and function determination in non-model species is to combine PacBio- and second generation sequencing methods [19,20]. *Sonneratia apetala* Buch.-Ham. is an excellent species with strong adaptability and a fast growth rate, which makes it a preferred species for artificial afforestation in difficult site during mangrove restoration programs [21]. However, almost no studies are available about the genome or the transcriptom of *S. apetala*, specially for Root tissues. Roots are the first organs that directly come in contact with the soil, they sense many abiotic stresses and allow adaptive adjustments. Understanding the effects of salt stress on the root system would provide insights into salt-tolerance mechanisms in mangroves [22]. In the present study, we utilized PacBio sequencing to generate a full-length transcriptome of *S. apetala*. To explore the underlying mechanism in salinity response, we identified DEGs in *S. apetala* roots that were salt-stressed as compared to the controls, and analysed their biological functions. We also combined mRNA and miRNA sequencing to explore the interactions between them. To our knowledge, this is the first report on the transcriptome profiling of *S. apetala* roots. This study provides a comprehensive understanding of salt response in *S. apetala* and offers novel insights into molecule functions of saline stress response in mangroves.

## Materials and methods

### Plant materials and RNA extraction

*S. apetala* seeds were harvested from a mangrove coastal belt in Techeng Island (21°09′ ~ 21°10′ N, 110°25′ ~ 110°27′ E), Guangdong, China. All the seeds were sown on an artificial soil in seedbeds to grow for about 70 days. Seedlings with a height of 10–16 cm were transplanted into polythene bags and 1-year-old uniformly developed seedlings were selected for salt stress treatments (or respective controls). Salinity stress was induced by applying 300 mmol/L NaCl every 3 days for a period of 14 days, whereas the control plants were treated with only water. Three biological replicates, RCK_a, RCK_b, RCK_c and RT_a, RT_b, RT_c, for control and salt treatment, respectively, were used. Root samples were collected, snap frozen in liquid nitrogen and then kept at  − 80°C until RNA extraction. Total RNA from each sample was extracted using a RNeasy Plant mini kit (Qiagen, Hilden, Germany) according to the manufacturer’s instructions. The quality of RNA was checked using Nanodrop 2000 (Thermo Scientific) and Agilent 2100 Bioanalyzer (Agilent Technologies, CA, United States). Only extractions with an RNA integrity value greater than 7.0 and OD260/280 ratio of 1.8–2.2 were used further.

### PacBio Iso-Seq library preparation and full‑length transcriptome analysis

To obtain the comprehensive full-length transcriptome of *S. apetala*, equal amounts of total RNA from the six replicates were pooled and used for Iso-Seq library preparation. PCR amplification was conducted to generate the cDNAs. The first stand cDNA was produced after reverse transcription of the mixed RNA sample using the SMARTer™ PCR cDNA synthesis kit, which was subsequently used as template for a large-scale PCR to synthesize double-stranded cDNA. The amplified cDNA was then cleaned up, SMRT dumbbell-shaped adapters were connected, and used for SMRTbell library construction using the PacBio template prep kit (Pacific Biosciences of California, Inc., California, United States). The established libraries were then validated and quantified using the Qubit 3.0 Fluorometer (Invitrogen, Carlsbad, United States). Finally, the qualified full cDNA library (with a concentration of > 10 ng/μl) was run using the binding kit 2.1 from the PacBio Sequel System at the Beijing Genomics Institute (BGI), China. SMRTlink v6.0 software and the Iso-Seq pipeline were used to process the raw Iso-Seq data. Raw reads were processed into error-corrected reads of inserts (ROIs) with the following parameters of minimum full pass > 1 and prediction accuracy > 0.85. The ROIs were classified into circular consensus sequences (CCS) and nonCCS subreads based on the presence or absence of sequencing adapters. CCS subreads with a minimum length of 300 bp were classified into full-length non-chimeric (FLNC) or non-full-length reads (NFL) based on whether the 5’ primer, 3’ primer, and poly-A tail were all observed. Iterative clustering for error correction (ICE) was applied to all the full-length sequences to obtain de novo consensus isoforms based on the sequence similarity. The Quiver quality-aware algorithm was used for error correction and high-quality full-length transcripts were classified with a criteria post-correction accuracy of above 99%. Finally, redundancies were removed using Cluster Database at High Identity with Tolerance (CD-HIT) to obtain final set of full-length isoforms, which were used as a reference for further analysis.

### Transcriptome sequencing and data analysis

RNA samples from the control and salinity treatments were used for mRNA sequencing. A total of six mRNA-seq libraries were constructed following the methods described by Li et al. [23]. Libraries were then sequenced on a BGISEQ-500 sequencing platform [24] to generate 150 bp paired-end (PE) reads. Raw reads were filtered and trimmed using SOAPnuke software (v1.5.2) [25] by removing the reads with a percentage of low-quality bases > 50%, reads with > 10% ‘N’ bases, reads with adapters, and trimmed reads shorter than 75 bp. All the clean reads were mapped to the full-length transcriptome obtained from PacBio Iso-Seq as a reference sequence using Bowtie 2 (v2.2.5) [26]. The Trinity software (v2.0.6) was used for assembly generation and transcript levels were determined through RSEM [27]. The transcript isoform level and gene-level counts were estimated using FPMK (fragments per kilobases per million reads) method. The DESeq2 [28] was used to identify DEGs between control and salinity treatments. Significant DEGs were selected with cut-off of *p* < 0.001, *Q* < 0.01 and a minimum two-fold change. Kyoto Encyclopedia of Genes and Genomes (KEGG) enrichment pathway analysis was performed with the phyper function in the R software package based on a corrected Q value of 0.05. For Gene Ontology (GO) analysis of DEGs, transcript sequences were mapped to the SwissProt database using the blastx function in Diamond [29]. Each transcript isoforms that annotated by SwissProt was then associated with GO terms using idmapping datasets (ftp.pir.georgetown.edu/databases/idmapping/idmapping.tb.gz). GO annotation was constructed for *S. apetala* using the AnnotationForge package in Bioconductor and GO enrichment analyses were performed with the help of clusterProfiler package in Bioconductor.

### Small RNA sequencing and data analysis

Six small RNA-seq libraries were constructed according to a previously described procedure [30]. Small RNA fragments of 10–30 nt length were isolated from total RNAs by using agarose gel electrophoresis. Libraries were then sequenced on a BGISEQ-500 platform to generate 50 bp single-end (SE) reads. Raw reads were filtered and trimmed to obtain clean sequencing reads after discarding the contaminations and low quantity reads with the assistance of SOAPnuke software (v1.5.2) [25]. The clean RNA reads were BLASTed to the sRNA databases with the help of AASRA [31] and cmsearch [32] to identify and remove rRNAs, snoRNAs, snRNAs and tRNAs. To identify known miRNAs, the clean reads were first screened against the full-length transcriptome reference sequences as well as matched to miRBase (http://www.mirbase.org/). Novel miRNAs were predicted using miRA [33] software. The abundance of miRNA transcripts was estimated by transcripts per million (TPM) and differentially accumulating miRNAs for each pair (RT vs. RCK) were identified on basis of the normalized TPM counts. miRNAs with |log_2_Fold change. normalized| ≥ 1, and Q-value <0.05 were used as cut-off to identify significantly differentially expressed miRNAs. Two types of software, psRobot [34] and TargetFinder [35] were used to predict the target genes of miRNAs and the intersection of their outputs was determined as the final result. Pathway annotation analysis for miRNA target genes was performed through screening KEGG Pathway databases (https://www.genome.jp/kegg/).

### Quantification of microRNA and target transcript levels with real-time quantitative polymerase chain reaction

cDNAs were obtained by reverse transcription of total RNA from six *S. apetala* samples (RCK_a, RCK_b, RCK_c, RT_a, RT_b, and RT_c). RT-qPCRs were performed on a CFX Connect™ Real-Time PCR Detection System through SYBR green-based real-time PCR. For determining miRNA expression levels, the miRNA RT-qPCR detection kit (Aidlab, Beijing, China) was adopted with miRNA specific forward primer and universal reverse primer (Table S1). The PCR samples were incubated at 94°C for 3 min, and 40 cycles of 20 s at 94°C, and 60°C for 40 s. For the miRNA target genes, gene-specific primers were designed with the assistance of Primer Premier v5 software (Table S1). RT-qPCR analysis was conducted with Light Cycler-FastStart DNA Master SYBR Green (Roche Applied Science, United States) following the manufacturer’s instructions. The PCR program included an initial denaturation at 94°C for 5 min, followed by 40 cycles of 30 s at 94 °C, 30 s at 57 °C and 30 s at 72 °C. The 2^–ΔΔCt^ method was used to calculate the abundance of each gene against U6 [36] and 18S RNA of *S. apetala* (GenBank number KJ161168) for miRNA and target genes, respectively. All reactions were run in triplicate to ensure reproducibility and reliability.

## Results

### Construction and annotation of a full‑length transcriptome of S. apetala

**Figure 1.**
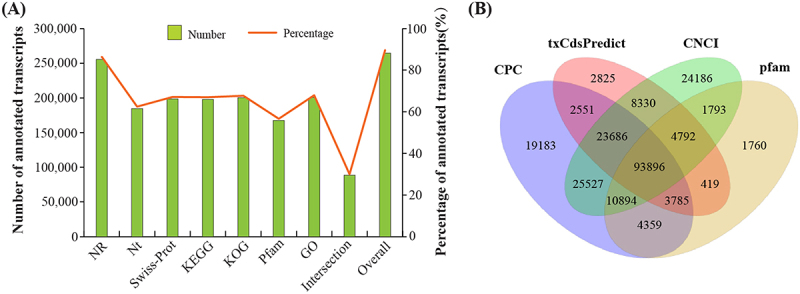
Functional annotation (A) and prediction of coding ability (B) of *S. apetala* transcripts.

For the construction of a full-length transcriptome, a pooled RNA sample was sequenced on a PacBio sequencing platform (Iso-Seq). It generated 868,540 polymerase and 15,629,500 subreads, respectively (Table 1). After quality control, a total of 776,450 circular consensus sequences (CCS) were obtained, with an average length of 4,209 bp. All CCS reads were classified into 1,702,463 full-length non-chimeric sequences (FLNC). These FLNCs were further clustered and polished to produce 1,552,748 high-quality consensuses. After removing redundant sequences, we finally obtained 295,501 transcript isoforms, with a mean length of 1,418 bp and N50 of 1,797 bp. Of these, a total of 264,709 transcripts (89.58%) were successfully annotated by searching public protein databases including NR, NT, GO, Swissprot, KEGG, KOG, and Pfam (Figure 1(a)). Of the annotated transcripts, 200,768 (67.94%) and 197,960 (66.99%) were annotated with the GO and KEGG databases, respectively. Moreover 88,678 (30.01%) transcripts were simultaneously annotated in all the seven databases (Figure 1(a)). To distinguish mRNAs and long non-coding RNAs (lncRNAs), the CPC [37], txCdsPredict, CNCI software [38] and pfam database [39] were used to predict the coding ability of transcripts. Annotation of a transcript as a mRNA or a lncRNA was confirmed when the outcomes of prediction of 3 of the 4 tools were same. This was, a total of 137,053 transcripts were confirmed as mRNAs (Figure 1(b)).

**Table 1.** Statistics of full-length transcriptome sequence data.

Category	Polymerase reads	Subreads	CCS	FLNC	Polished consensus	PRPC
number	868,540	15,629,500	776,450	1,702,463	1,552,748	295,501
mean length (bp)	61221	3,332	4,209	1,205	1,204	1,418

CCS, circular consensus; FLNC, full-length non-chimeric; PRPC, post- redundancy polished consensus.

### Analysis of mRNA and sRNA sequencing data

**Figure 2.**
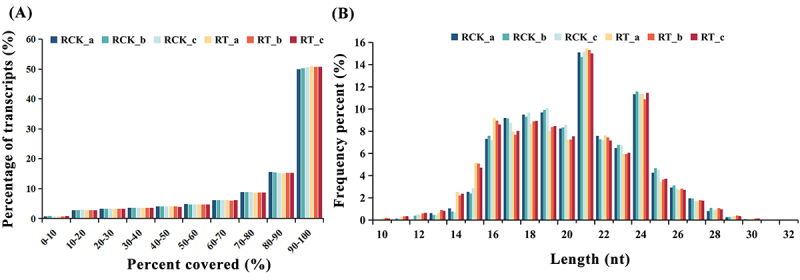
Coverage of reads (%) for transcripts in the roots of *S. apetala* (A) and the size distribution of small RNA sequences in different libraries (B). RCK, control; RT, salinity treatments. A, B, C represents the three repetitions. nt, nucleotides.

Overall, for BGISEQ-500 RNA-seq, more than 260 Mb raw reads were generated from six sequenced libraries of *S. apetala* roots (Table 2). After filtering low-quality raw reads and removing rRNAs, each sample had an averaged 43.1 Mb high-quality paired-end reads, with Q20 and Q30 values of more than 96.8% and 91.3%, respectively. Filtered reads were aligned and mapped to the *S. apetala* full-length transcriptome sequence. A mapping rate of 87.1% to 88% was observed, and the reads were evenly distributed across various regions of the transcript (Figure 2(a)). All the samples showed a coverage of 90–100% for more than 50% transcripts. A Pearson’s correlation coefficient with a minimum value of 0.872 was observed for the abundance of mRNAs between replicates (Table S2). To further confirm the accuracy of our sequencing data, we performed principal component analysis (PCA) based on gene expression features. The results showed that among six samples, RCK prominently separated from the RT and no outliers existed for biological replicates (Figure S1a). These results indicated that the quality of sequencing data was highly satisfactory and could be used for the subsequent analyses.

**Table 2.** Summary of RNA-seq datasets for six samples along with QC.

Sample	Total rawreads (Mb)	Total cleanreads (Mb)	Total cleanbases (Gb)	Clean readsQ20 (%)	Clean readsQ30 (%)	Clean readsratio (%)	Total mapping (%)	Uniquelymapping (%)
RCK_a	43.78	43.07	6.46	97.11	92.81	98.38	87.62	5.67
RCK_b	43.75	43.13	6.47	96.92	92.03	98.58	87.59	5.69
RCK_c	43.81	43.08	6.46	96.83	91.92	98.33	87.05	5.71
RT_a	43.76	43.15	6.47	96.89	91.96	98.61	88.03	5.7
RT_b	43.79	43.11	6.47	97.26	93.02	98.45	87.73	5.67
RT_c	43.79	43.21	6.48	97.33	93.12	98.68	87.69	5.66

CCS, circular consensus; FLNC, full-length non- chimeric; PRPC, post- redundancy polished consensus.

sRNA deep sequencing from *S. apetala* roots grown in NaCl-free (RCK) and NaCl treated conditions yielded a total of 189.2 million raw reads. After low-quality tags, adapters contaminants, poly (A) sequences and reads smaller than 18 nt were removed, 159.6 million clean sRNAs were acquired with an average Q20 value of 98.7%. An alignment to the *S. apetala* full-length transcriptome sequence for each library revealed over 80.1% clean sRNAs were successfully matched. Table 3 shows an overview of the raw sRNA-seq, high-quality and clean reads with quality filtering for each sequence library. The pearson’s correlation coefficient between repetitions were equal to or greater than 0.832 (Table S3) and outliers were not discovered through PCA (Figure S1B). All of these shows a high quality of the sequencing data for sRNA analysis. The distributions of sRNA lengths were similar among the six sRNA-seq libraries (Figure 2(b)). For all the six libraries, the most abundant class was of 21 nt sRNAs, whereas 24 nt sRNAs represented the second most frequent length, making up an average of approximately 15.1% and 11.3% of all sRNAs in the six libraries, respectively.

**Table 3.** Sample-wise statistics for data filtering and removing of adapters from sRNA sequencing.

Sample	Raw tag count	Low-quallity tag count	Invalid adapter tag count	PolyA tag count	Short valid length tag	Clean tag count	Q20 of clean tag (%)	Percentage of clean tag (%)	Mapped tag	Percentage of Mapped tag (%)
RCK_a	31674322	463281	437892	133	4336528	26436488	98.7	83.46	21183514	80.1
RCK_b	32467265	523437	476538	165	3233942	28233183	99	86.96	22935460	81.2
RCK_c	29346278	556037	498176	127	3220956	25070982	98.6	85.43	21360378	85.2
RT_a	32096237	700238	943652	652	4222319	26229376	98.8	81.72	21656370	82.6
RT_b	31589272	673542	582439	145	3923615	26409531	98.6	83.60	21948637	83.1
RT_c	32069531	538257	503216	152	3830182	27197724	98.6	84.81	22762423	83.7

Clean tags mean clean sRNAs.

### Differential expression of mRNAs for S. apetala exposed to salinity

**Figure 3.**
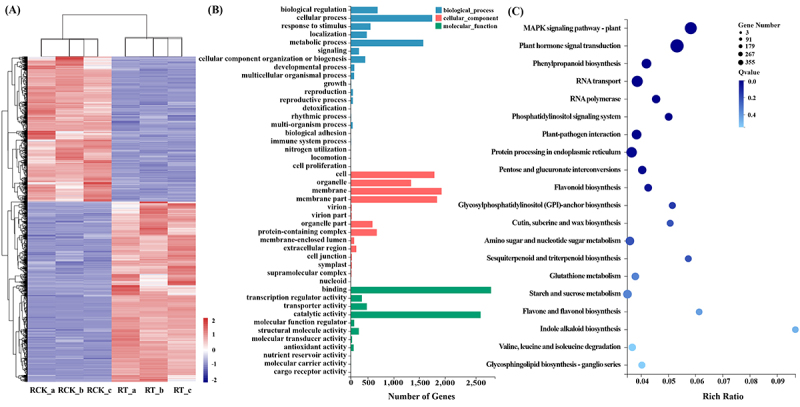
Genes differentially expressed between the control (RCK) and the saline treated (RT) roots. (A) Hierarchical clustering of 6,686 DEGs on the basis of their log_2_FPKM values. Red and blue indicate the transcripts with increased and decreased levels, respectively. A, B and c represent the three biological replicates for each treatment. (B) GO annotation for differentially expressed genes. Vertical coordinates indicated the secondary classification of gene ontologies; horizontal coordinates represented the number of related genes. Different colors indicate the three categories of gene ontology terms. (C) KEGG pathway enrichment analysis of differentially expressed genes. The vertical and horizontal axis represented KO names and the ‘rich factor’ of the identified pathway, respectively. The color of the circles corresponded to the significance level of the enrichment, and the size of the circles represent the number of differentially expressed genes within a pathway.

To identify DEGs under salt stress, RNA-seq clean reads were mapped to the reference transcriptome generated by SMRT sequencing and fragments per kilobase of transcript per million base pairs (FPKM) counts were compared. A total of 6,686 transcripts were differentially expressed (*p* < 0.001, *Q* < 0.01), of which 3,705 and 2,981 genes were up-/down-regulated in NaCl treated *S. apetala* roots as compared to NaCl-free (RCK) samples (Data S1, S2). Of these differentially expressed genes, 1,405 showed a tissue-specific expression under different growth conditions ((Data S2). Furthermore, expression-based clustering classified all the DEGs into two sub-clusters. The first sub-cluster contained genes that were down-regulated in the NaCl-free (RCK) roots but up-regulated in NaCl treated roots, and vice-versa for the second sub-cluster (Figure 3(a); Data S2). These DEGs were involved in signalling transduction, transcription, transport, metabolism, and miscellaneous processes. Amonst transcription factors, four WRKY genes (isoform_221382, isoform_40773, isoform_220602, and isoform_209733) were differentially expressed in salt-treated roots; also an ethylene-insensitive protein (EIN3, isoform_222252) was induced in *S. apetala* roots under salt stress. Four TPS genes (isoform_283094, isoform_9274, isoform_24535 and isoform_265679) and a PP2C (isoform_11335; belonging to the phosphatase group) showed differential expression under salt-stress conditions. ABCC2 (isoform_240511), kup (isoform_230455) and PIP (isoform_74355) are a few examples of transporters that were up-regulated after salt stress. In addition, other genes related to metabolism and stress resistance were also sharply up-regulated by salt treatment.

To illustrate the function of the DEGs during salt stress response, GO terms were analysed, and all differentially regulated transcripts in the salt treated *S. apetala* roots were grouped into three categories of biological process, cellular component, and molecular function (Figure 3(b); Data S3). ‘Cellular process’ (1,545) represented the most dominant term amongst the biological process ontology; the dominant term for cellular component were ‘membrane part’ (1636); and ‘binding’ (2,660) represented the largest molecular function category. Furthermore, the results of KEGG enrichment revealed MAPK signalling pathway – plant (ko04016), plant hormone signal transduction (ko04075), phenylpropanoid biosynthesis (ko00940), RNA transport (ko03013) and RNA polymerase (ko03020) as the most probable candidates related to salt-stress response process of *S. apetala* (*p* < 0.01, *Q* < 0.05; Figure 3(c), Supplementary Datas S4, S5). Further, three signal transduction terms, ‘MAPK signaling pathway – plant’ (ko04016), ‘plant hormone signal transduction’ (ko04075), ‘phosphatidylinositol signaling system’ (ko04070), were found in the category of environmental information processing and thus indicating import roles in salt stress resistance. The majority of representatives of the ‘metabolism’ category were related to phenylpropanoid biosynthesis (ko00940; 168, 5.3%). Some other pathways were also significantly enriched, which included ‘phosphatidylinositol signaling system’ (ko04070), ‘plant-pathogen interaction’ (ko04626) and ‘protein processing in endoplasmic reticulum’ (ko04141), indicating their possible involvement during response to salt stress.

### Identification of known and novel miRNAs

**Figure 4.**
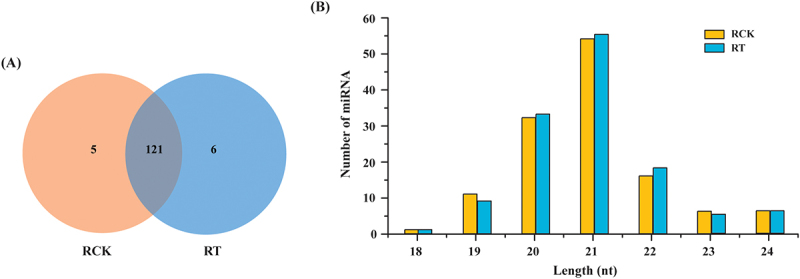
miRNAs identified in *S. apetala* by high-throughput sequencing. (A) Distribution of miRNAs in control (RCK) and 14 days-salinity treated (RT) plants. (B) The length distribution of *S. apetala* miRnas. A, B and C represent the three biological replicates for each treatment. nt, nucleotides.

After removing rRNA, snoRNA, snRNA, tRNA and other sncRNAs, the remaining sequences were aligned to those in miRBase v. 22.1 database to identify the miRNAs conserved in *S. apetala*. Then, the unannotated unique sRNA sequences were retained and utilized to predict putative novel miRNA candidates. In total, 113 known miRNAs, belonging to 27 miRNA-families, along with 19 predicted novel miRNAs, were identified (Figure 4(a), Supplementary Data S6, S7). Of these, 107 known and 19 novel miRNAs were especially found in the control samples, whereas 111 and 16 miRNAs, respectively, were found in the salt treated roots (Figure 4(a), Supplementary Data S6, S7). Most miRNAs (121; 91.7% of the total) were detected in both of the two different groups (RCK and RT). Five miRNAs (Sap-miR166c, Sap-miR5368, Sap-nmiR4, Sap-nmiR5 and Sap-nmiR11) were uniquely detected in RCK library, whereas six known miRNAs, namely, Sap-miR169r-3p, Sap-miR172a_1, Sap-miR394a_2, Sap-miR395p-3p, Sap-miR8051-5p and Sap-miR828-3p, were specifically found in the RT libraries. The length of identified miRNAs varied from 18 nt to 24 nt, where 21-nt and 20-nt long miRNAs accounted for the two major sizes classes (Figure 4(b)). Among the known miRNA families, 18 known miRNA families contain more than one member; where the two largest families were MIR319 (11 members), and MIR396 (10 members). Also, several conserved miRNA families, such as MIR395, MIR399, MIR4995 MIR530, MIR5368, MIR6300, MIR8051, MIR827 and MIR828, had just one member (Supplementary Data S6). The precursor of the 19 newly predicted novel miRNAs varied from 110-nt to 284-nt in length, which was consistent with the general length of pre-miRNAs (Supplementary Data S7). Furthermore, a typical hairpin structure of the precursors, with detected miRNA star sequences (miRNA*), was regarded as a confirmation for the potential novel miRNAs (Figure S2; Supplementary Data S7).

### Expression of miRNAs and their target genes in response to salt stress

**Figure 5.**
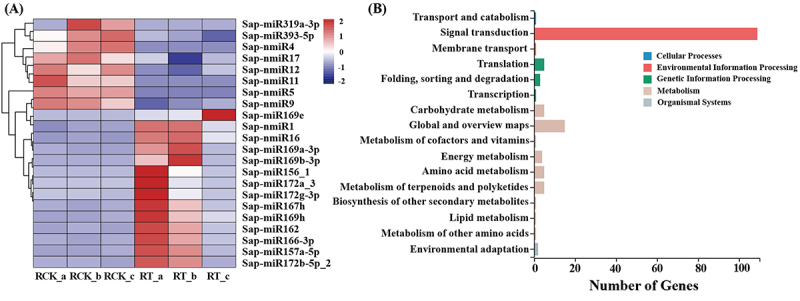
Differentially accumulated *S. apetala* miRNAs after 14 days of salt treatment (A) and and KEGG annotation of their target genes (B). RCK, control; RT, salinity treatment. A, B and c represented the three biological replicates for each treatment. Vertical and horizontal coordinates in (B) indicate the secondary classification of the KEGG terms and the number of related genes within a pathway, respectively.

To investigate the effect of salinity of *S. apetala* miRNAs, differentially expressed (DE) miRNAs were identified with the help of log_2_ (RT/RCK), using a cut-off of an absolute value > 1 and a Q-value <0.05. In total, 22 DE miRNAs were identified, which included 14 (12 known and 2 novel) up-regulated and 8 (2 known and 6 novel) down-regulated miRNAs (Figure 5(a); Supplementary Data S8). The expression patterns of the DE miRNAs were similar to those of the DEGs, with the number of up regulated miRNAs more than that of down-regulated. Several known miRNA families, such as MIR169 and MIR172, related to the response to NaCl conditions were differentially increased [4]. Moreover, three novel miRNAs, Sap-nmiR4, Sap-nmiR5 and Sap-nmiR11 were strongly differentially repressed, with their log_2_ |RT/RCK| values being > 5 in salt stressed samples.

Using the psRobot [34] and TargetFinder tools [35], a total of 411 target genes were predicted for 17 salt-responsive miRNAs (14 known and 3 novel), which corresponded to 524 miRNA-target regulatory interactions (466 for conserved and 58 for novel miRNAs; Supplementary Data S9). Pathway annotation analysis for the target genes revealed 138 annotations from the KEGG database (Figure 5(b); Supplementary Data S10). The majority of the pathways for the target genes were related to ‘environmental information processing’ (110); of which 109 were categorize under ‘signal transduction’ and and 1 was annotated for ‘membrane transport’. One hundred six targets were related to ‘plant hormone signal transduction’ (ko04075), which was the highest among all KO terms. Also, three target genes, isoform_74467, isoform_180254 and isoform_225634, were annotated to be a part of ‘MAPK signaling pathway – plant’ (ko04016), and thus were related to ‘signal transduction’ during salt stress resistance. These results indicate that most miRNAs participate in salt stress-resistance modulation by the regulation of signal transduction pathways in *S. apetala*. Additionally, isoform_107695 that associated with ‘ABC transporters’ (ko02010), represented the only pathway under ‘membrane transport’.

### Integrated and regulation analysis of Co-expressed miRNAs-target

**Figure 6.**
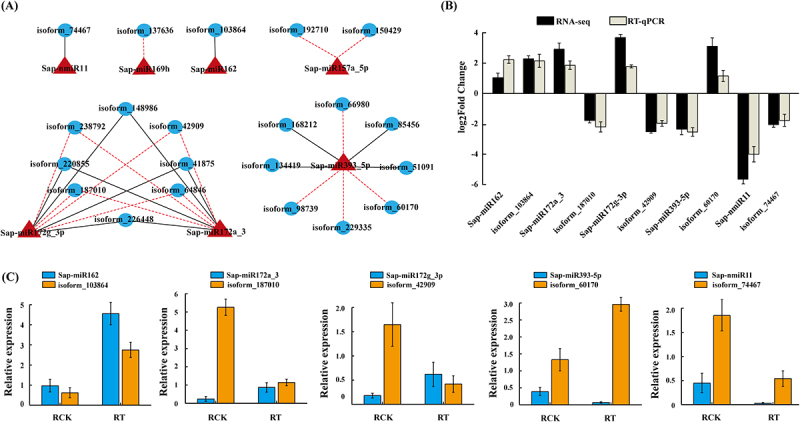
Co-expressed and RTqPCR validation of miRNA-target pairs in control (RCK) and 14 days (RT) salinity treated *S. apetala*. (A) A proposed regulatory network of related miRNA-target pairs. The black solid and the red dashed lines represent the positive and the negative correlations, respectively. miRNAs and targets are marked by triangle and circles, respectively. (B) The expression profiles of miRnas, as obtained by real-time quantitative polymerase chain reaction (RT-qPCR) and by deep sequencing are shown. Five miRNAs and their target genes were randomly selected from the co-expression network for RT-qPCR validation studies. (C) Relative transcript abundances of the 5 miRNA-target pairs as obtained with the help of RT-qPCR in control (RCK) and salt treated samples (RT).

To discover co-altered miRNA-target interactions pairs during salt stress, we also focused on the alteration patterns of 411 miRNA-target genes. Of these, 21 putative targets of seven miRNAs were significantly differentially accumulated after salt treatment (Figure 6(a); Supplementary Data S11). The expression correlation (*r*) between differentially expressed miRNAs and differentially expressed mRNAs was evaluated using the Pearson Correlation Coefficient (PCC), which exhibited an absolute value of more than 0.33 (*p* = 0.047), suggesting the potential regulatory relationships of miRNAs and their targets during response to salt stress. Subsequently, the co-expressed interaction networks were constructed the between the differentially expressed miRNAs and their target genes that were differentially expressed. We obtained 29 miRNA-target pairs, 15 and 14 of which showed a negative and a positive interaction, respectively (Figure 6(a); Supplementary Data S11). A miRNA (for example, the up-regulated Sap-miR172a_3 and Sap-miR172g-3p) was co-expressed with up to 8 target genes. Simultaneously, Sap-miR157a-5p exhibited negative correlations with two target gene, isoform_192710 and isoform_150429 after a salt treatment. The down-regulated isoform_74467, which was paired to Sap-nmiR11, encode for an endochitinase protein, and is associated with both ‘MAPK signaling pathway-plant’ (ko04016) and ‘metabolic pathways’ (ko01100) in our KEGG annotation (Supplementary Data S10). Sap-miR393-5p targeted eight target genes that encode an F-box family protein TIR1, which is annotated to participate in ‘signal transduction’ during salt stress response. Interestingly, APETALA2-like (AP2) is predicted to be regulated by both Sap-miR172a_3 and Sap-miR172g-3p. As a specific transcription factor for plants, AP2 plays a key regulatory role in various stress response processes. These results indicate that miRNAs were involved in multiple aspects of *S. apetala* root response to salt stress, which include signal transduction and metabolism.

The expression levels of 10 randomly selected miRNAs and mRNAs from the co-expressed miRNA-target interaction network were further validated using RT-qPCR. The RT-qPCR results support the deep sequencing profiles (Figure 6(b)). For instance, Sap-miR172a_3 was induced under salt stress conditions, while its target gene, isoform_187010 (AP2), was repressed. On the other hand, the expressions of Sap-nmiR11 and its target counterpart, isoform_74467 (CHIB), were decreased in salt-stressed roots of *S. apetala* (Figure 6(c)). These results show that the high-throughput sequencing data were reproducible and reliable, confirming the authenticity of miRNA and implied that the expression of miRNA would be adjusted for *S. apetala* to adapt salt stress condition. The co-expressed miRNA-mRNA interaction network also provide a valuable clue for probing the complex regulatory mechanism of salt response in *S. apetala.*

## Discussion

### Strategies of S. apetala to resist salt stress

The response to salt stress in plants is accompanied by a broad range of intracellular processes, including stress perception, signal transduction, transcription, and protein biosynthesis [2]. As mangrove plants grow in saline environments for a long time, they have evolved special mechanism to tolerate it as compared to plants that grow in terrestrial fresh waters. *S. apetala* is a pioneering mangrove species, which plays a crucial role in keeping the ecological balance of a coastal zone [21]. Currently, there are almost no genomic and transcriptomic resources for *S. apetala*, which has been a major barrier to investigate the functions and regulations of genes related to salt stress adaptation of *S. apetala*. To date, limited number of studies are available about the complex mechanism of salt stress response of *S. apetala*. In the past, short-read RNA-seq technology has provide a convenient tool for estimating mRNA expression patterns under various stress conditions [16]. However, short-read RNA-seq is not always sufficient for constructing a reliable transcriptome, especially for species that do not have reference genomes. Third-generation SMRT sequencing from PacBio platform can produce full-length transcript sequences without assembly [17]. Here, we established a full-length transcriptome of *S. apetala* using PacBio Iso-Seq, and further explored the changes in gene expression patterns under saline conditions with the help of short-read RNA-seq. This experimental design produced high-quality transcripts and reliable estimates of changes in transcript accumulation. For instance, more than 85% of RNA-seq reads could be successfully matched to the reference transcriptome (Table 2). We found a high-degree correlation, of a minimum value of 0.859 (Table S2) and 0.832 (Table S3) respectively, between different biological replicates of gene expression for mRNAs and sRNAs. Also, PCA based on transcriptomic profile revealed a clear separation between samples of RCK and RT with no outliers discovered for all individuals (Figure S1). All of these ensured the quality and accuracy of the sequencing data for further analyses and gene expression comparisons. Moreover, we successfully identified key genes and the underlying pathways involved in salt stress response process of *S. apetala*. These data provide a deeper understanding of potential mechanisms of stress response at the molecular level in *S. apetala*.

### Characterization of salt stress-responsive mRNAs in S. apetala

By analysing the patterns of mRNA accumulations in two groups (RCK and RT) of *S. apetala* plants, we identified 6,686 differentially expressed transcripts (*p* < 0.001, *Q* < 0.01; Data S1, S2). Further, GO-based annotations of DEGs were analysed. ‘Binding’ (2,660), ‘membrane part’ (1636) and ‘cellular process’ (1,545) were the most abundant classifications of DEGs during salt stress response (Figure 3b; Data S3). KEGG analysis showed that DEGs were predominantly enriched in salt-related pathways such as ‘MAPK signaling pathway – plant’ (ko04016), ‘plant hormone signal transduction’ (ko04075) and ‘phenylpropanoid biosynthesis’ (ko00940).

We have identified genes that are possibly involved in salt stress response process. Twenty seven genes that encode stress-related transcription factors were identified [40], of which 4, 9 and 14 belong to the WRKY, bZIP and AP2/EREBP family, respectively (Table S4; Supplementary Data S2). The WRKY proteins are a superfamily of transcription factors that are widely distributed in many plant species, some of which are a principal regulator of salinity responses [41]. WRKYs collectively play a part in modulating salt responses, which include hormone signal transduction, ion transport response, osmotic balance and antioxidant response [41]. WRKY TFs bind W-box, trigger and activate their downstream salt-resistant genes. Also, WRKYs serves as inhibitory factors of hormone signalling, causing reduction to salinity in plants. Three down-regulated isoforms of WRKY2 (isoform_221382, isoform_40773, and isoform_209733) and an up-regulated isoform of WRKY22 (isoform_220602) exhibited significant alteration in their expressions after salt treatment (Supplementary Data S2). Particularly, these WRKYs were categorized into the ‘environmental adaptation’ category, and are involved in ‘plant-pathogen interaction’ (ko04626) and ‘MAPK signaling pathway – plant’ (ko04016) (Supplementary Data S4). These findings implied that down-regulation of the transcriptional repressor WRKY2 reduces salt sensitivity in *S. apetala*, and that up-regulation of the W-box-bound WRKY22 initiates the transcription of salt-related genes, thereby increasing salt tolerance of *S. apetala*.

HD-Zip is a unique Homogenic domain (HB) transcription factor, which contains a homology domain (HD) motif and a leucine zipper (LZ) motif [42]. HD-Zip’s can efficiently regulate plants’ response to various environmental signals [42]. Abiotic stresses lead to the overproduction of reactive oxygen species (ROS) in plants that could cause damage to carbohydrates, lipids, proteins and DNA and ultimately result in oxidative stress [43]. In *Betula alba*, BpbZIP1 act as significant ROS scavenging enzymes, reduces the levels of intracellular ROS accumulation, and hence improve the tolerance to salt stresses [44]. Additionally, in NaCl- and abscisic acid (ABA)-treated cotton, GhHB1 increase significantly at an early stage, and then they decrease sharply [42]. The over-expression of PsnHDZ63 could confer salt tolerance in transgenic poplar, *Populus simonii* × *P. nigra* [42]. In our study, 9 isoforms encoding HD-Zip proteins were significantly altered, which imply their potential participation in the response to salt and osmotic stresses.

Further, we also identified some genes encoding enzymes or functional proteins, such as DELLAs, type 2C phosphatase 2C proteins (PP2Cs), SNF1-related protein kinases 2 (SNRK2), mitogen-activated protein kinases (MAPKs), ATP-binding cassette (ABCs) type transporters, calcium-binding protein CMLs and basic endochitinases B (CHIBs) (Table S4; Supplementary Data S2). These proteins are directly or indirectly implicated in plant salt stress responses. Hormones mediated regulation plays a pivotal role in mounting of adaptive responses of plant to adverse environmental signals, including salinity stress [45]. Specially, a probable cross-talk between hormones signalling underlying salinity stress response has been constructed by Nongpiur *et al*. (2016) [46]. DELLA proteins are crucial regulators that guide the cross-talk of various phytohormones and integrate plant responses to environmental cues [45]. Previous studies have demonstrated that the accumulation of DELLA proteins restrain the growth of plants and enhance resistance to stresses [45]. DELLA proteins could act as positive regulators of salinity stress response by inhibiting the negative JAZ protein regulators during salinity response [46], or through interaction with XERICO, an inducer of ABA biosynthesis [46]. Herein, three isoforms, isoform_113410, isoform_264356 and isoform_13710, encoding DELLA proteins exhibited increased accumulation, and four (isoform_86291, isoform_14532, isoform_3363 and isoform_3826) were decreased under saline condition. The various alteration of expression on DELLA proteins indicated their complex mechanisms during the regulation of adaptation to salinity in *S. apetala*.

Several components, including the ABA receptors PYR/PYL/RCARs, the PP2C negative regulators and the SnRK2 positive regulators, constitute a double negative modulatory model during ABA signalling [47,48]. In response to environmental stimulus, ABA signal triggers the interaction between PYR/PYL/RCAR and PP2C, which result in PP2C inhibition and SnRK2 activation [49]. The function of PP2Cs and SnRK2 in ABA-mediated modulation of abiotic stressful cues such as salinity have now been confirmed [47,49]. In *A. thaliana*, PP2C-type phosphatases have been sub-divided into 13 groups (A – H) [49]. ABI1, ABI2, HAB1, HAB2, AHG1 and PP2CA/AHG3 are the most prominent members in Group A, which have been defined as negative regulators of ABA early signal transduction [47]. In abi2–1 mutants of *A. thaliana*, the interaction between ABI2 and salt overly sensitive 2 (SOS2) was interrupted, which resulted in an improvement of salt stress resistance and ABA insensitivity [48]. However, the overexpression of a group G PP2C gene, AtPP2CG1, enhanced the salt-resistance of *A. thaliana* [49]. Similarly, a wheat PP2C gene (TaPP2C1; a group F2 subfamily PP2C) could confer resistance to salt stress by inducing an ABA-independent antioxidant system in transgenic tobacco [49]. The SnRK2 family consists of the ABA-activated protein kinases SnRK2.2, SnRK2.3, SnRK2.6, SnRK2.7, and SnRK2.8, and the ABA-independent subclass 1 protein kinases SnRK2.1, SnRK2.4, SnRK2.5, SnRK2.9, and SnRK2.10 [50]. Previous studies have demonstrated that the heterologous overexpression of poplar PtSnRK2.5 and PtSnRK2.7 genes increased salt stress tolerance in *A. thaliana* [51]. Further, the Snf1-related protein kinases SnRK2.4 and SnRK2.10, are involved in the modulation of ROS homoeostasis [52] and maintenance of root system architecture during salt stress of *A. thaliana* [50]. In the present study, expression of six *PP2Cs* and a *SNRK2* were altered under saline conditions, which is a reminiscent of their involvement in salinity adaptation response of *S. apetala* (Table S4; Supplementary Data S2).

Further, the MAPK class of serine/threonine protein kinases are a part of conserved signalling pathways, and these are involved in transfer of external stimuli to the cellular organization [53]. MAPK cascades comprise of three sequential phosphorylation and activation components, a MAP kinase kinase kinase (MEKK/MAPKKK), a MAP kinase kinase (MKK/MAPKK), and a MAP kinase (MPK/MAPK) [54]. MAPKs phosphorylate transcription factors, protein kinases and transporters [54,55]. Signalling through MAPK cascades lead to a wide variety of cellular responses, including response to various stresses [53]. The involvement of MAPK in salt stress induced signal transduction has been reported in multiple plant specials [54]. For example, AtMPK4 and AtMPK6 can be activated by AtMEKK1 (MAPKKK) when *A. thaliana* was exposed to cold and salt stress [56]. In alfalfa, salt stress enhances the activation of MAPK (SIMK), which is mediated by a MAPKK homolog, SIMKK [56,57]. Three MAPKs, ZmMPK3, ZmMAPK5, and ZmSIMK1 are triggered by salt stress in *Zea mays* [56]. The salt-stress mediated MAPK activation was also reported in cotton [54], rice, cucumber, and tobacco [56]. In our study, 15 members of MAPK cascade including MEKK1, MAPKKK17/18, ANP1, MKK4, MPK1, MPK3, MPK4 and MPK6, exhibited significant changes in their accumulation under salinity condition (Table S4; Supplementary Data S2); of these, 8 were induced by salinity stress. KEGG annotations classified all the MAPKs into the category of ‘environmental information processing’ (Supplementary Data S4), further confirming their potential important roles involved in MAPK cascades during the salt stress response of *S. apetala*. However, the specific gene members and mechanisms underlying MAPK cascade system remain to be further investigated in *S. apetala* response to salinity.

### Integration analysis of miRNAs and mRNA during response to salt stress

miRNAs are well-known regulators of gene expression in plants and they play crucial roles in a variety of abiotic stress responses [58]. In this study, a comprehensive investigation was carried on miRNA-functions during the salt stress response of *S. apetala* by using deep sequencing. A total of 113 known miRNAs, belonging to 27 families, and 19 novel miRNAs were detected from the six sRNA libraries (Figure 4(a), Supplementary Data S6, S7), among which 22 miRNAs were differentially accumulated (Figure 5(a); Supplementary Data S8). These included miR156, miR169, miR172 and miR393, which are the major salt stress-regulated miRNAs in plants [59], and it indicate a common thread of miRNA-mediated salt stress adaptation across different specials. Identification of other conserved and novel miRNAs during salt stress in *S. apetala* (Figure 5(a); Supplementary Data S8) is novel into this study. miRNA members belonging to the same family showed consistent expression pattern after salt stress treatment in *S. apetala*. This result indicates that miRNAs in the same family may play similar roles in response to salt stress. Although different species of plants respond to stress by utilizing various miRNA-guided regulation, studies have demonstrated that hub miRNAs, such as of miR169, miR319, miR393, miR396, and miR398, could be involved in coping up with multiple stresses, such as drought, salinity, and cold [60,61]; these studies further indicate that the targets of these miRNA are associated with sensing stress signals and other stimuli. For instance, miR169 has been defined to be implicated in various stresses including salt stress [62], water deficit, drought, cold and nitrogen-starvation stresses [63], fungal and bacterial infection [64]; miR319 function as a bridge, linking plant responses to ABA, drought and salt stress [65,66]. The fine-tuning of miRNA accumulation during various stresses indicate the presence of a shared regulatory mechanism across distinct stress responses, which also implies for the existence of common targets that are modulated by these miRNAs across responses to various abiotic and biotic stresses. On the other hand, the expression of miR393 was reported to be induced under conditions of salt stress in *Arabidopsis*, rice, and a grass species [67]. However, in this study, we observed that one member of miR393 family, Sap-miR393-5p, was repressed under saline stress in *S. apetala*. These results revealed a difference of the same miRNA for salt stress response across different plants specials, probably due to the species specific in binding to target mRNAs, attempting to reveal complex miRNA-mediated gene regulation was involved in adaptive responses towards stresses. The dissimilar expression trends observed in miR319 in different species indicate that their targets may play varied roles in different regulatory pathways in response to salt stress, which make it necessary to analyse the miRNAs of specific plant species under salt stress.

A total of 411 target genes were predicted for 17 significantly differentially accumulated miRNAs, which comprised of 524 miRNA-target pairs (Supplementary Data S9). KEGG pathways analysis classified ‘signal transduction’ as a predominant pathway under the category of ‘environmental information processing’ (Figure 5(b); Supplementary Data S10), which indicted that these pathways may probably promote high salt tolerance of *S. apetala*. Also, ‘global and overview maps’, ‘carbohydrate metabolism’, ‘amino acid metabolism’, ‘translation’ and other pathways were activated under salt stress, which possibly might initiate the production of salt stress-related metabolites, and potentially act as crucial modulators during processes such as ion homoeostasis, oxidation protection and ROS signalling. When we focused on the accumulation patterns of the 411 miRNA targets under saline condition, we found that seven miRNAs and their 21 predicted targets (29 miRNA-target pairs) were co-expressed (correlation |*r*| ≥ 0.33, *p* < 0.05) after salt treatment (Figure 6(a); Supplementary Data S11). Among these miRNA-target related gene pairs, 14 were positively correlated, suggesting that positive correlation is similar to the negative correlation. These results indicate that complex relationship networks exist between miRNAs and their targets and point towards other regulatory mechanisms than co-transcription under salt stress in *S. apetala*.

Salt stress is known to be regulated by the cross-talk of phytohormones, with auxin having been described as a key mediator of modulating a balance during changes of signalling, biosynthesis, ion transport, and finally mounting of physiological response patterns [68]. miR393, which targets F-box genes that encode auxin receptors, is closely related to various biotic and abiotic stresses [67]. The miR393-TIR1 regulatory module module has multiple functions that manipulate the responsiveness to of plants salt stress via the auxin pathway [67]. In *Arabidopsis*, the overexpression of a miR393-resistant TIR1 gene (mTIR1) clearly enhanced salt stress tolerance due to an increased osmoregulation and Na^+^ exclusion [69]. Within the miRNA-target co-expressed interactions in our study, four of eight target genes, isoform_60170, isoform_134419, isoform_229335 and isoform_66980, are annotated to code for TIR1, and exhibited an increased expression under salt stress. On the other hand, the accumulation of their miRNA regulator, Sap-miR393a, was down-regulated after salt treatment (Figure 6(a); Supplementary Data S11). We propose that the decreased expression of Sap-miR393a attenuate miRNA-mediated mRNA cleavage, resulting in increased levels of the target gene TIR1 protein at the translational level, thereby activating and enhancing an up-regulation of auxin signalling to alter salt resistance of *S. apetala*.

Further, two of the conserved miRNAs, Sap-miR172a_3 and Sap-miR172g-3p, exhibited cross-functionality with the AP2 targets. miR172/AP2 module has been defined as multifaceted, which is involved in orchestrating salt tolerance in plants [70]. In the study of Pan *et al*., (2016) [71], miR172 was shown to regulate salt tolerance by repressing the AP2/EREBP-type TF gene, salt suppressed AP2 domain-containing (SSAC1), and acts as a long-distance stress-related signal. These observations indicate that Sap-miR172a_3/Sap-miR172g-3p-targeting AP2 May exhibit critical functions during the *S. apetala* survival under saline conditions. Further, novel miRNAs also appear to play a critical role in regulating signal transduction. For instance, isoform_74467, which could encode CHIB, is a predicted target for Sap-nmiR11, and was associated with MAPK pathway in our KEGG annotation (Supplementary Data S10). The increased expression of genes involved in MAPK signaling pathway could result in induction of signaling pathways, activating responsive targets for adaptation to salt stress of plants. Herein, both of Sap-nmiR11 and CHIB exhibited down-regulated expression, and this positively correlated miRNA-target counterpart suggests that complex regulatory networks exist between miRNAs and their targets in *S. apetala* response to salt stress. Such observations indicate a pivotal role of novel miRNAs in salt tolerance of *S. apetala* and the newly identified miRNA may serve as a supplementary resource for salt-tolerance miRNA mining. We acknowledge that due to a lack of genome resources for *S. apetala*, we were not able to annotate the targets of Sap-miR157a-5p and Sap-miR162, both of which were up regulated under saline condition (Supplementary Data S8). Zhang et al., [72] and Parmer *&* Shaw [73] have shown that miR157 and miR162 family members are also induced in salt stressed sesame and maize. These results encourage us to hypothesize a potential crucial role of Sap-miR157a-5p and Sap-miR162 during the process of salinity tolerance in *S. apetala*. Further functional studies, such as those involving bioinformatics and transgenic analyses, would help to shed light regulation of adaptive response to salinity by these two unknown miRNAs.

## Conclusions

In summary, we integrated SMRT sequencing, RNA-seq and miRNA-seq to construct a comprehensive transcriptome of *S. apetala* roots. We obtained an overview of the transcriptomic landscape during the process of salt response of *S. apetala*. We explored the potential mechanism of salt stress adaptations by investigating the accumulation of miRNAs and mRNAs between control and salt stressed plants. By constructing of a regulatory network of differentially accumulated miRNAs and their target genes, we identified valuable miRNA-mRNA pairs and their potential role in salt stress modulation. The results of this study provide a global view of mRNA and miRNA regulation during the salt stress response. The key regulators identified in this study exhibited significant alterations in their levels after salt treatment and provide information on candidates for the further characterization of the molecular mechanisms underlying salinity response in *S. apetala*.

## Supplementary Material

Supplementary Table S4.docx

Supplementary Table S1.docx

Supplementary Table S2.docx

Supplementary Table S3.docx

Supplementary_Data (1).xlsx

Supplementary Figure S1.png

Supplementary Figure S2.jpg

## Data Availability

The sequence data reported in this study have been deposited into the Genome Sequence Archive (https://ngdc.cncb.ac.cn/gsa/), Chinese Academy of Sciences with the accession number CRA006866 and CRA006863.
