# Supplementary material for: miRNA–mRNA integrated analysis reveals candidate genes associated with salt stress response in Halophytic Sonneratia apetala
Source: RNA Biol. 2025 Apr 28;22(1):1–13. doi: 10.1080/15476286.2025.2496097 (PMC12045576; doi:10.1080/15476286.2025.2496097)
Supplement: Supplementary Table S4.docx [file KRNB_A_2496097_SM0188.docx]

**Table S4.** Functional annotation of differentially expressed genes that are involved in response to salt stress.

| Gene ID | Abbreviation of gene | Annotated description | Salt-related pathway |
| --- | --- | --- | --- |
| isoform_176407 | MEKK1 | mitogen-activated protein kinase kinase kinase 1 [EC:2.7.11.25] | signaling transduction (MAPK signaling) |
| isoform_178639 | MEKK1 | mitogen-activated protein kinase kinase kinase 1 [EC:2.7.11.25] | signaling transduction (MAPK signaling) |
| isoform_250353 | ABA2 | [xanthoxin dehydrogenase [EC:1.1.1.288]](https://www.kegg.jp/entry/1.1.1.288) | signaling transduction (hormone signaling) |
| isoform_275116 | ABCC1 | [ATP-binding cassette, subfamily C (CFTR/MRP), member 1 [EC:7.6.2.3]](https://www.kegg.jp/entry/7.6.2.3) | ion homeostasis |
| isoform_240511 | ABCC2 | ATP-binding cassette, subfamily C (CFTR/MRP), member 2 | ion homeostasis |
| isoform_248081 | ANP1 | [mitogen-activated protein kinase kinase kinase ANP1 [EC:2.7.11.25]](https://www.kegg.jp/entry/2.7.11.25) | signaling transduction (MAPK signaling) |
| isoform_265909 | ANP1 | [mitogen-activated protein kinase kinase kinase ANP1 [EC:2.7.11.25]](https://www.kegg.jp/entry/2.7.11.25) | signaling transduction (MAPK signaling) |
| isoform_280524 | ANP1 | [mitogen-activated protein kinase kinase kinase ANP1 [EC:2.7.11.25]](https://www.kegg.jp/entry/2.7.11.25) | signaling transduction (MAPK signaling) |
| isoform_148986 | AP2 | AP2-like factor, euAP2 lineage | signaling transduction (hormone signaling) |
| isoform_42909 | AP2 | AP2-like factor, euAP2 lineage | signaling transduction (hormone signaling) |
| isoform_41875 | AP2 | AP2-like factor, euAP2 lineage | signaling transduction (hormone signaling) |
| isoform_64846 | AP2 | AP2-like factor, euAP2 lineage | signaling transduction (hormone signaling) |
| isoform_226448 | AP2 | AP2-like factor, euAP2 lineage | signaling transduction (hormone signaling) |
| isoform_187010 | AP2 | AP2-like factor, euAP2 lineage | signaling transduction (hormone signaling) |
| isoform_220855 | AP2 | AP2-like factor, euAP2 lineage | signaling transduction (hormone signaling) |
| isoform_238792 | AP2 | AP2-like factor, euAP2 lineage | signaling transduction (hormone signaling) |
| isoform_281397 | AP2 | AP2-like factor, euAP2 lineage | signaling transduction (hormone signaling) |
| isoform_192839 | BAK1 | brassinosteroid insensitive 1-associated receptor kinase 1 [EC:2.7.10.1 2.7.11.1] | signaling transduction (MAPK signaling, hormone signaling) |
| isoform_245451 | BIN2 | [protein brassinosteroid insensitive 2 [EC:2.7.11.1]](https://www.kegg.jp/entry/2.7.11.1) | signaling transduction (hormone signaling) |
| isoform_147952 | CALM | calmodulin | signaling transduction (Ca2+-dependent signal transduction, MAPK signaling, hormone signaling) |
| isoform_74467 | CHIB | basic endochitinase B [EC:3.2.1.14] | signaling transduction (MAPK signaling, hormone signaling) |
| isoform_10029 | CHIB | basic endochitinase B [EC:3.2.1.14] | signaling transduction (MAPK signaling, hormone signaling) |
| isoform_106097 | CHIB | basic endochitinase B [EC:3.2.1.14] | signaling transduction (MAPK signaling, hormone signaling) |
| isoform_172261 | CHIB | basic endochitinase B [EC:3.2.1.14] | signaling transduction (MAPK signaling, hormone signaling) |
| isoform_204119 | CHIB | basic endochitinase B [EC:3.2.1.14] | signaling transduction (MAPK signaling, hormone signaling) |
| isoform_21816 | CHIB | basic endochitinase B [EC:3.2.1.14] | signaling transduction (MAPK signaling, hormone signaling) |
| isoform_235205 | CHIB | basic endochitinase B [EC:3.2.1.14] | signaling transduction (MAPK signaling, hormone signaling) |
| isoform_2403 | CHIB | basic endochitinase B [EC:3.2.1.14] | signaling transduction (MAPK signaling, hormone signaling) |
| isoform_289367 | CHIB | basic endochitinase B [EC:3.2.1.14] | signaling transduction (MAPK signaling, hormone signaling) |
| isoform_294689 | CHIB | basic endochitinase B [EC:3.2.1.14] | signaling transduction (MAPK signaling, hormone signaling) |
| isoform_4114 | CHIB | basic endochitinase B [EC:3.2.1.14] | signaling transduction (MAPK signaling, hormone signaling) |
| isoform_4846 | CHIB | basic endochitinase B [EC:3.2.1.14] | signaling transduction (MAPK signaling, hormone signaling) |
| isoform_56908 | CHIB | basic endochitinase B [EC:3.2.1.14] | signaling transduction (MAPK signaling, hormone signaling) |
| isoform_61089 | CHIB | basic endochitinase B [EC:3.2.1.14] | signaling transduction (MAPK signaling, hormone signaling) |
| isoform_71327 | CHIB | basic endochitinase B [EC:3.2.1.14] | signaling transduction (MAPK signaling, hormone signaling) |
| isoform_82761 | CHIB | basic endochitinase B [EC:3.2.1.14] | signaling transduction (MAPK signaling, hormone signaling) |
| isoform_229765 | clpB | ATP-dependent Clp protease ATP-binding subunit ClpB | protein turnover |
| isoform_241071 | CML | calcium-binding protein CML | signaling transduction (Ca2+-dependent signal transduction, MAPK signaling, hormone signaling) |
| isoform_71444 | CML | calcium-binding protein CML | signaling transduction (Ca2+-dependent signal transduction, MAPK signaling, hormone signaling) |
| isoform_186172 | CNGC | cyclic nucleotide gated channel, plant | ion homeostasis |
| isoform_102452 | copA, ctpA, ATP7 | P-type Cu+ transporter [EC:7.2.2.8] | ion homeostasis |
| isoform_67469 | CPK | calcium-dependent protein kinase [EC:2.7.11.1] | signaling transduction (hormone signaling) |
| isoform_86291 | DELLA | DELLA protein | signaling transduction (hormone signaling) |
| isoform_14532 | DELLA | DELLA protein | signaling transduction (hormone signaling) |
| isoform_113410 | DELLA | DELLA protein | signaling transduction (hormone signaling) |
| isoform_264356 | DELLA | DELLA protein | signaling transduction (hormone signaling) |
| isoform_13710 | DELLA | DELLA protein | signaling transduction (hormone signaling) |
| isoform_3363 | DELLA | DELLA protein | signaling transduction (hormone signaling) |
| isoform_3826 | DELLA | DELLA protein | signaling transduction (hormone signaling) |
| isoform_209819 | EBF1_2 | EIN3-binding F-box protein | signaling transduction (hormone signaling), reactive oxygen species (ROS) |
| isoform_117204 | EIF3A | translation initiation factor 3 subunit A | Protein turnover |
| isoform_188151 | EIF3A | translation initiation factor 3 subunit A | Protein turnover |
| isoform_222252 | EIN3 | ethylene-insensitive protein 3 | signaling transduction (hormone signaling), reactive oxygen species (ROS) |
| isoform_486 | ER | LRR receptor-like serine/threonine-protein kinase ERECTA [EC:2.7.11.1] | signaling transduction (MAPK signaling) |
| isoform_173239 | EREBP | EREBP-like factor | signaling transduction (hormone signaling) |
| isoform_90793 | EREBP | EREBP-like factor | signaling transduction (hormone signaling) |
| isoform_2438 | EREBP | EREBP-like factor | signaling transduction (hormone signaling) |
| isoform_213550 | EREBP | EREBP-like factor | signaling transduction (hormone signaling) |
| isoform_54565 | EREBP | EREBP-like factor | signaling transduction (hormone signaling) |
| isoform_33608 | FLS | flavonol synthase [EC:1.14.20.6] | signaling transduction (MAPK signaling) |
| isoform_199458 | FLS2 | [LRR receptor-like serine/threonine-protein kinase FLS2 [EC:2.7.11.1]](https://www.kegg.jp/entry/2.7.11.1) | signaling transduction (MAPK signaling) |
| isoform_28222 | FLS2 | [LRR receptor-like serine/threonine-protein kinase FLS2 [EC:2.7.11.1]](https://www.kegg.jp/entry/2.7.11.1) | signaling transduction (MAPK signaling) |
| isoform_281052 | GAPA | glyceraldehyde-3-phosphate dehydrogenase (NADP+) (phosphorylating) [EC:1.2.1.13] | basic metabolism |
| isoform_67349 | GH3 | auxin responsive GH3 gene family | signaling transduction (hormone signaling) |
| isoform_144245 | GH3 | auxin responsive GH3 gene family | signaling transduction (hormone signaling) |
| isoform_275188 | GH3 | auxin responsive GH3 gene family | signaling transduction (hormone signaling) |
| isoform_264688 | HD-ZIP | homeobox-leucine zipper protein | signaling transduction (hormone signaling) |
| isoform_198124 | HD-ZIP | homeobox-leucine zipper protein | signaling transduction (hormone signaling) |
| isoform_271096 | HD-ZIP | homeobox-leucine zipper protein | signaling transduction (hormone signaling) |
| isoform_105093 | HD-ZIP | homeobox-leucine zipper protein | signaling transduction (hormone signaling) |
| isoform_17708 | HD-ZIP | homeobox-leucine zipper protein | signaling transduction (hormone signaling) |
| isoform_103747 | HD-ZIP | homeobox-leucine zipper protein | signaling transduction (hormone signaling) |
| isoform_267087 | HD-ZIP | homeobox-leucine zipper protein | signaling transduction (hormone signaling) |
| isoform_214867 | HD-ZIP | homeobox-leucine zipper protein | signaling transduction (hormone signaling) |
| isoform_246476 | HD-ZIP | homeobox-leucine zipper protein | signaling transduction (hormone signaling) |
| isoform_71818 | HSP20 | HSP20 family protein | protein turnover |
| isoform_255571 | KIN | DNA/RNA-binding protein KIN17 | signaling transduction (hormone signaling) |
| isoform_230455 | kup | KUP system potassium uptake protein | ion homeostasis |
| isoform_40549 | MAPKKK17_18 | mitogen-activated protein kinase kinase kinase 17/18 | signaling transduction (MAPK signaling) |
| isoform_164055 | MKK4_5 | mitogen-activated protein kinase kinase 4/5 [EC:2.7.12.2] | signaling transduction (MAPK signaling) |
| isoform_147089 | MKK4_5 | mitogen-activated protein kinase kinase 4/5 [EC:2.7.12.2] | signaling transduction (MAPK signaling) |
| isoform_279549 | MKS1 | MAP kinase substrate 1 | signaling transduction (MAPK signaling) |
| isoform_39621 | MKS1 | MAP kinase substrate 1 | signaling transduction (MAPK signaling) |
| isoform_293101 | MPK1_2 | mitogen-activated protein kinase 1/2 [EC:2.7.11.24] | signaling transduction (MAPK signaling) |
| isoform_168045 | MPK3 | mitogen-activated protein kinase 3 [EC:2.7.11.24] | signaling transduction (MAPK signaling) |
| isoform_240542 | MPK3 | mitogen-activated protein kinase 3 [EC:2.7.11.24] | signaling transduction (MAPK signaling) |
| isoform_164531 | MPK3 | mitogen-activated protein kinase 3 [EC:2.7.11.24] | signaling transduction (MAPK signaling) |
| isoform_182097 | MPK4 | mitogen-activated protein kinase 4 [EC:2.7.11.24] | signaling transduction (MAPK signaling) |
| isoform_6115 | MPK4 | mitogen-activated protein kinase 4 [EC:2.7.11.24] | signaling transduction (MAPK signaling) |
| isoform_289248 | MPK6 | mitogen-activated protein kinase 6 [EC:2.7.11.24] | signaling transduction (MAPK signaling) |
| isoform_243533 | MYC2 | transcription factor MYC2 | signaling transduction (MAPK signaling) |
| isoform_51752 | MYC2 | transcription factor MYC2 | signaling transduction (MAPK signaling) |
| isoform_99419 | NCED | 9-cis-epoxycarotenoid dioxygenase [EC:1.13.11.51] | signaling transduction (hormone signaling) |
| isoform_74355 | PIP | aquaporin PIP | ion homeostasis |
| isoform_200892 | plc | phospholipase C [EC:3.1.4.3] | signaling transduction (hormone signaling) |
| isoform_180163 | PLCD | phosphatidylinositol phospholipase C, delta [EC:3.1.4.11] | signaling transduction (hormone signaling) |
| isoform_27088 | PLCD | phosphatidylinositol phospholipase C, delta [EC:3.1.4.11] | signaling transduction (hormone signaling) |
| isoform_267947 | PP2C | protein phosphatase 2C [EC:3.1.3.16] | signaling transduction (MAPK signaling) |
| isoform_23532 | PP2C | protein phosphatase 2C [EC:3.1.3.16] | signaling transduction (MAPK signaling) |
| isoform_181080 | PP2C | protein phosphatase 2C [EC:3.1.3.16] | signaling transduction (MAPK signaling) |
| isoform_11335 | PP2C | protein phosphatase 2C [EC:3.1.3.16] | signaling transduction (MAPK signaling) |
| isoform_18212 | PP2C | protein phosphatase 2C [EC:3.1.3.16] | signaling transduction (MAPK signaling) |
| isoform_236637 | PP2C | protein phosphatase 2C [EC:3.1.3.16] | signaling transduction (MAPK signaling) |
| isoform_67401 | RBOH | respiratory burst oxidase [EC:1.6.3.- 1.11.1.-] | signaling transduction (hormone signaling) |
| isoform_178550 | RBOH | respiratory burst oxidase [EC:1.6.3.- 1.11.1.-] | signaling transduction (hormone signaling) |
| isoform_271081 | RGLG | [E3 ubiquitin-protein ligase RGLG [EC:2.3.2.27]](https://www.kegg.jp/entry/2.3.2.27) | signaling transduction (hormone signaling) |
| isoform_240960 | SAUR | SAUR family protein | signaling transduction (hormone signaling) |
| isoform_245193 | SNF1 | carbon catabolite-derepressing protein kinase [EC:2.7.11.1] | signaling transduction (hormone signaling) |
| isoform_29293 | SNF1 | carbon catabolite-derepressing protein kinase [EC:2.7.11.1] | signaling transduction (hormone signaling) |
| isoform_246385 | SNRK2 | serine/threonine-protein kinase SRK2, SNF1-related protein kinases 2 [EC:2.7.11.1] | signaling transduction (hormone signaling) |
| isoform_57983 | SPCH | transcription factor SPEECHLESS | signaling transduction (MAPK signaling) |
| isoform_66980 | TIR1 | transport inhibitor response 1 | protein turnover, morphological adaption, signaling transduction (hormone signaling) |
| isoform_85456 | TIR1 | transport inhibitor response 1 | protein turnover, morphological adaption, signaling transduction (hormone signaling) |
| isoform_51091 | TIR1 | transport inhibitor response 1 | protein turnover, morphological adaption, signaling transduction (hormone signaling) |
| isoform_60170 | TIR1 | transport inhibitor response 1 | protein turnover, morphological adaption, signaling transduction (hormone signaling) |
| isoform_229335 | TIR1 | transport inhibitor response 1 | protein turnover, morphological adaption, signaling transduction (hormone signaling) |
| isoform_98739 | TIR1 | transport inhibitor response 1 | protein turnover, morphological adaption, signaling transduction (hormone signaling) |
| isoform_134419 | TIR1 | transport inhibitor response 1 | protein turnover, morphological adaption, signaling transduction (hormone signaling) |
| isoform_168212 | TIR1 | transport inhibitor response 1 | protein turnover, morphological adaption, signaling transduction (hormone signaling) |
| isoform_24535 | TPS | trehalose 6-phosphate synthase/phosphatase [EC:2.4.1.15 3.1.3.12] | osmotic protection |
| isoform_265679 | TPS | trehalose 6-phosphate synthase/phosphatase [EC:2.4.1.15 3.1.3.12] | osmotic protection |
| isoform_283094 | TPS | trehalose 6-phosphate synthase/phosphatase [EC:2.4.1.15 3.1.3.12] | osmotic protection |
| isoform_9274 | TPS | trehalose 6-phosphate synthase/phosphatase [EC:2.4.1.15 3.1.3.12] | osmotic protection |
| isoform_285495 | UBC | ubiquitin C | ion homeostasis |
| isoform_289087 | UBC | ubiquitin C | ion homeostasis |
| isoform_169426 | USP | UDP-sugar pyrophosphorylase [EC:2.7.7.64] | ion homeostasis, basic metabolism adaption |
| isoform_232602 | VIP1 | transcription factor VIP1 | signaling transduction (MAPK signaling) |
| isoform_209733 | WRKY2 | WRKY transcription factor 2 | signaling transduction (MAPK signaling) |
| isoform_221382 | WRKY2 | WRKY transcription factor 2 | signaling transduction (MAPK signaling) |
| isoform_40773 | WRKY2 | WRKY transcription factor 2 | signaling transduction (MAPK signaling) |
| isoform_220602 | WRKY22 | WRKY transcription factor 22 | signaling transduction (MAPK signaling) |
