# Supplementary material for: miRNA–mRNA integrated analysis reveals candidate genes associated with salt stress response in Halophytic Sonneratia apetala
Source: RNA Biol. 2025 Apr 28;22(1):1–13. doi: 10.1080/15476286.2025.2496097 (PMC12045576; doi:10.1080/15476286.2025.2496097)
Supplement: Supplementary Table S1.docx [file KRNB_A_2496097_SM0187.docx]

**Table S1.** Primer sequences used for real-time PCR analysis of miRNAs and their corresponding targets.

| Gene ID | | Forward primer（5′→3′） | Reverse primer （5′→3′） |
| --- | --- | --- | --- |
| Sap-miR162 | | TCGATAAACCTCTGCATCCGG |  |
| Sap-miR172a_3 | | AGAATCTTGATGATGCTGCAT |  |
| Sap-miR172g-3p | | GGAATCTTGATGATGCTGCAG |  |
| Sap-miR393-5p | | TTCCAAAGGGATCGCATTGAT |  |
| Sap-nmiR11 | | CGGCGAGAGCGGGTCGCCGC |  |
| isoform_51121 | | CTTAGACTCTCTCTCTCTTTGT | TCAGAAGATATAACAGCGGTA |
| isoform_77358 | | GATCGTGGATAGTCAGCACA | TCTCGACTGTTGTGCTGACT |
| isoform_12563 | | ACTCACTCATCTGCTCTCTCTC | AGAGAGAGAGAGAGAGAGCAG |
| isoform_227383 | | TGTGAAGACAGAGGGTAGA | TCTGTCTGTCTCTCTCTACCC |
| isoform_214947 | | TCTACAAGAGATACTGTGACC | GTCACAGTATCTCTTGTAGA |
| Universal reverse primer for miRNA | |  | GTGCAGGGTCCGAGGT |
| U6 | | CCTTCGGGGACATCCGATAAAA | GCAGGGGCCATGCTAATCTTCT |
| 18s rRNA | CCGCCTCTGGTGTGCACCGGTC | | CCCCCGGAACCCAAGGACTTTG |
