# Supplementary material for: miRNA–mRNA integrated analysis reveals candidate genes associated with salt stress response in Halophytic Sonneratia apetala
Source: RNA Biol. 2025 Apr 28;22(1):1–13. doi: 10.1080/15476286.2025.2496097 (PMC12045576; doi:10.1080/15476286.2025.2496097)
Supplement: Supplementary Table S2.docx [file KRNB_A_2496097_SM0186.docx]

**Table S2.** Pearson correlation analysis of gene expression patterns revealed by RNA-seq across various samples.

| sample | RCK_a | RCK_b | RCK_c | RT_a | RT_b | RT_c |
| --- | --- | --- | --- | --- | --- | --- |
| RCK_a | 1 | 0.9854 | 0.9852 | 0.8681 | 0.8590 | 0.8660 |
| RCK_b | 0.9854 | 1 | 0.9886 | 0.8717 | 0.8692 | 0.8726 |
| RCK_c | 0.9852 | 0.9886 | 1 | 0.8769 | 0.8753 | 0.8778 |
| RT_a | 0.8681 | 0.8717 | 0.8769 | 1 | 0.9883 | 0.9924 |
| RT_b | 0.8590 | 0.8692 | 0.8753 | 0.9883 | 1 | 0.9927 |
| RT_c | 0.8660 | 0.8726 | 0.8778 | 0.9924 | 0.9927 | 1 |

Note: RCK, control sample; RT, samples treated with salt for 14 d. a, b, c represent the three replicates.
