# Supplementary material for: miRNA–mRNA integrated analysis reveals candidate genes associated with salt stress response in Halophytic Sonneratia apetala
Source: RNA Biol. 2025 Apr 28;22(1):1–13. doi: 10.1080/15476286.2025.2496097 (PMC12045576; doi:10.1080/15476286.2025.2496097)
Supplement: Supplementary Table S3.docx [file KRNB_A_2496097_SM0185.docx]

**Table S3.** Pearson correlation analysis of sRNA abundances as revealed by deep sequencing across all the samples.

| Sample | RCK_a | RCK_b | RCK_c | RT_a | RT_b | RT_c |
| --- | --- | --- | --- | --- | --- | --- |
| RCK_a | 1 | 0.9566 | 0.9657 | 0.8320 | 0.8547 | 0.9627 |
| RCK_b | 0.9566 | 1 | 0.9851 | 0.8553 | 0.8764 | 0.9478 |
| RCK_c | 0.9657 | 0.9851 | 1 | 0.8618 | 0.8811 | 0.9408 |
| RT_a | 0.8320 | 0.8553 | 0.8618 | 1 | 0.9882 | 0.8724 |
| RT_b | 0.8547 | 0.8764 | 0.8811 | 0.9882 | 1 | 0.8980 |
| RT_c | 0.9627 | 0.9478 | 0.9408 | 0.8724 | 0.8980 | 1 |

Note: RCK, controls; RT, samples treated with salt for 14 d. a, b, c represent the three replicates.
