# Supplementary figures and images for: miRNA–mRNA integrated analysis reveals candidate genes associated with salt stress response in Halophytic Sonneratia apetala
Source: RNA Biol. 2025 Apr 28;22(1):1–13. doi: 10.1080/15476286.2025.2496097 (PMC12045576; doi:10.1080/15476286.2025.2496097)

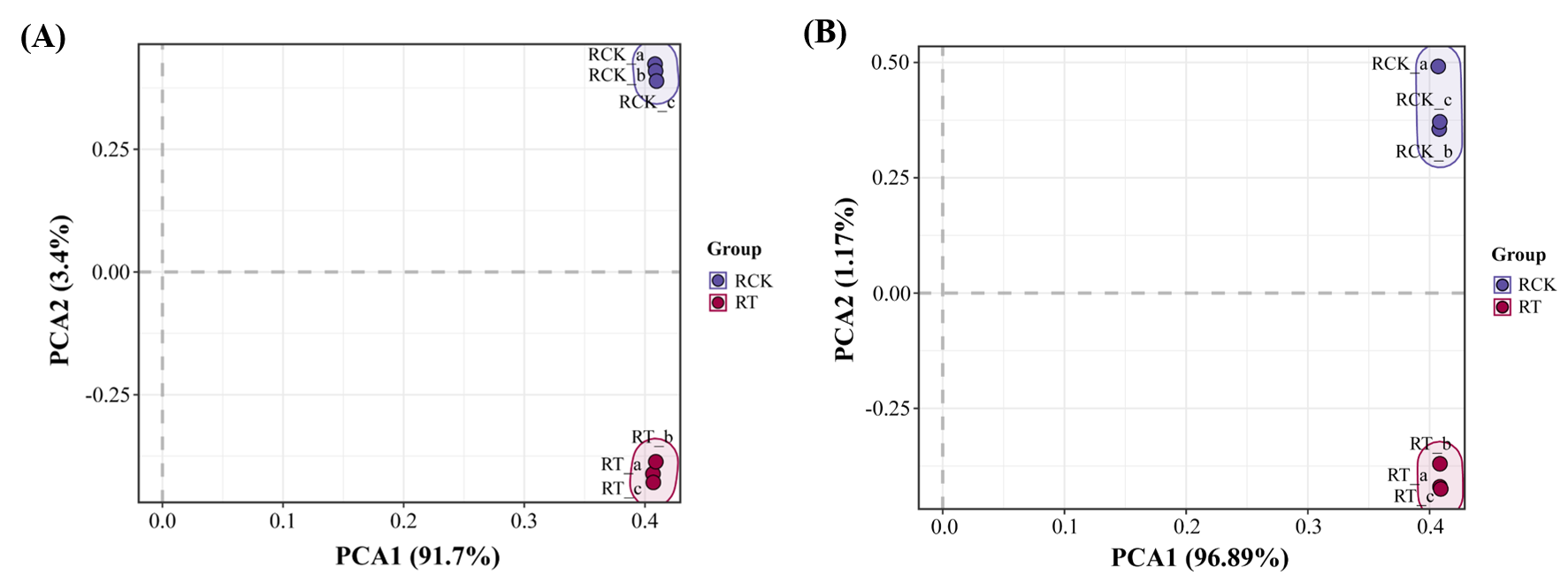

Supplement: Supplementary Figure S1.png [file KRNB_A_2496097_SM0183.png]

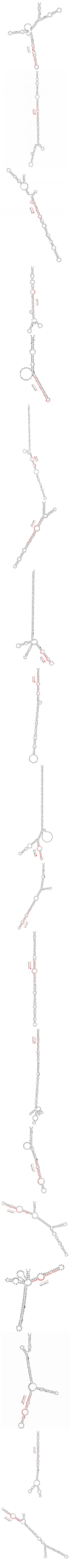

Supplement: Supplementary Figure S2.jpg [file KRNB_A_2496097_SM0182.jpg]
